# Dual-delivery of FGF-2/CTGF from Silk Fibroin/PLCL-PEO Coaxial Fibers Enhances MSC Proliferation and Fibrogenesis

**DOI:** 10.1038/s41598-017-08226-0

**Published:** 2017-08-17

**Authors:** Ruodan Xu, Huiling Zhao, Hanif Muhammad, Mingdong Dong, Flemming Besenbacher, Menglin Chen

**Affiliations:** 1 Department of Engineering, Aarhus University, DK-8000 Aarhus C, Denmark; 2 Interdisciplinary Nanoscience Center (iNANO), Aarhus University, DK-8000 Aarhus C, Denmark

## Abstract

The success of mesenchymal stem cell transplantation is highly dependent on their survival and controlled fate regulation. This study demonstrates that dual-delivery of connective tissue growth factor (CTGF) and fibroblast growth factor 2 (FGF-2) from a core-shell fiber of Silk Fibroin/poly(L-lactic acid-co-ε-caprolactone)-polyethylene oxide (SF/PLCL-PEO) enhanced fibrogenic lineage differentiation of MSCs. The core-shell structure was confirmed by transmission electron microscopy (TEM), fluorescence microscopy and attenuated total reflection (ATR) Fourier transform infrared (FTIR) spectroscopy. A sequential release of FGF-2 and CTGF was successfully achieved in this manner. FGF-2 plays an important role in stem cell proliferation and, meanwhile when accompanied with CTGF, has a slightly additive effect on fibrogenic differentiation of MSCs, whereas CTGF promotes fibrogenesis and alleviates osteogenesis, chondrogenesis and adipogenesis.

## Introduction

Mesenchymal stem cells (MSCs) can be efficiently isolated from adult bone marrow, are capable of extended proliferation and hold the potential to differentiate into mesenchymal lineages including osteoblasts, chondrocytes, and adipocytes^1^. It has also been suggested that they can modulate the host immune responses when transplanted^2^. These characteristics make them an attractive candidate for biological cell-based tissue repair approaches^3^. However, MSCs survival and incorporation at the graft are still crucial issues for transplantations. Some cytokines have been identified to enhance the proliferation of MSCs, such as Platelet-derived growth factor (PDGF) through the activation of c-Jun N-terminal kinase (JNK) signaling^4^, Transforming growth factor β1 (TGFβ1) inducing the rapid nuclear translocation of β-catenin in a Smad3-dependent manner^5^. Fibroblast growth factors (FGFs) are a family of growth factors involved in many functions such as cell proliferation, migration, and differentiation; and are critically important in tissue development, maintenance and wound repair^6^. Previous studies have shown FGF-2 or basic fibroblast growth factor (b-FGF) to be able to increase rat, rabbit, canine and human MSCs proliferation and maintains their multilineage differentiation potential *during in vitro* expansion^7–10^.

Apart from the expansion, the prospect of controlling MSC differentiation is a crucial regulatory and clinical requirement. The differentiation of MSCs into mesenchymal lineage is known to be controlled by diverse transcription factors and signaling cascades such as Hedgehog^11^, NEL-like protein 1 (NELL-1)^12^ and β catenin-dependent Wnt^13^. In association with TGFβ1, insulin-like growth factor-I (IGF-I) and bone morphogenic protein 2 (BMP-2) were known to induce the differentiation of MSCs into chondrocytes^14^. Until recently, connective tissue growth factor (CTGF) or CCN2, a member of CCN family^15^ has been identified to sufficiently direct MSCs differentiation towards fibroblasts^16^ and promote connective tissue healing in a rodent injury model^17^. Our previous study^18^ and the study reported by Tong *et al*.^19^ both demonstrated CTGF loaded 3D scaffold was capable of fostering fibroblastic differentiation *in vitro*.

Half of the female population over the age of 50 experiences disorders of the pelvic floor caused by weakening and rupture of supportive connective tissue sheet or fascia^20^. This disorder can lead to pelvic organ prolapse (POP) seen as a herniation of the bladder, uterus or intestines into the vagina^21^. The lifetime risk for undergoing POP surgery is estimated to 11.1–19% with a risk of further reoperation up to 30%^22^. To this date, no efficient treatment without side effects or risk of recurrence is available. With the promise of controllable fibrogenic commitment of MSCs, treatment of pelvic floor disorders could be brought forward by deploying a biodegradable electrospun mesh to deliver therapeutic cells to enhance formation of new connective tissue that will take over mechanical load when the mesh degrades.

Considering FGF-2 and CTGF with individual functions on improving MSCs survival and proliferation and steering MSCs to a fibroblastic commitment, respectively, a model of dual delivery device at the wound region for combination treatments of FGF-2 and CTGF would draw attention for their synergistic effect on MSCs and broaden their clinical potential for pelvic floor connective tissue regeneration. Silk fibroin (SF) is a promising protein-based biomaterial for tissue engineering which has shown excellent mechanical properties, slow degradation, well biocompatibility and low inflammatory response compared to collagen and poly lactic acid^23^. SF has been extensively used in biomedical applications such as sutures, drug delivery matrices, and 3D scaffolds for ligament, bone, cartilage, fat, neural, cardiac, ocular, bladder and vasculature engineering^24^. Poly(L-lactic acid-co-ε-caprolactone) (PLCL) are a copolymer of L-lactic acid and ε-caprolactone whose mechanical properties and degradation rate can be controlled and changed by the relative composition of monomers^25^. PLCL nanofibers have been demonstrated to support the growth and proliferation of many cell types while showing inadequate cell affinity due to its hydrophobicity and the absence of recognition sites for cell adhesion^26^. Polyethylene oxide (PEO), well known to suppress protein adhesion^27^ and applied as anti-inflammatory polymeric coatings for implantable biomaterials and devices, has been added to improve the hydrophilicity and suppress protein adhesion.

In this study, coaxial electrospun SF/PLCL-PEO core-shell fibers were, for the first time, used to dual-deliver FGF-2 and CTGF in a temporally controlled manner for enhancing MSCs survival and fibrogenesis collectively. The fiber structure and wettability were characterized using Scanning Electron Microscopy (SEM), fluorescent microscopy, attenuated total reflection (ATR) Fourier transform infrared (FTIR) and contact angle measurement. The release profile of dual-delivery of FGF-2/CTGF was investigated using ELISA assay, and their synergistic effects on cell viability, proliferation and differentiation were analyzed using live-dead staining, lactate dehydrogenase test (LDH), cell counting kit-8 (CCK-8), real-time qPCR and immunocytochemistry staining.

## Materials and Methods

### Preparation of silk fibroin

Silk fibroin (SF) was extracted from cocoons of B. mori according to the protocol^28^. Briefly, 2.5 g silk cocoon pieces were added into 1.0 L boiling aqueous solution of 0.02 M Na_2_CO_3_ (Sigma-Aldrich) for 30 min. Then, the resultant silk fibroin was washed with Milli-Q ultrapure water for three times. After drying in a fume hood overnight, the degummed silk fibroin was weight and dissolved in a 9.3 M lithium bromide (LiBr, Sigma-Aldrich) solution (25% w/v) at 60 °C for 4–6 h. The solution was dialyzed against Milli-Q ultrapure using Slide-A-Lyzer dialysis cassette (Thermo Scientific). The final aqueous solution with the concentration of 7.2% w/v was used as the core solution.

### Preparation of Electrospinning Solution and Electrospinning of Coaxial Fibers

PLCL copolymer (70:30 lactic acid: caprolactone, Rosomer, Evonic) and poly(ethylene oxide)(PEO) (Sigma-Aldrich, St. Louis, USA) were dissolved in hexafluoroisopropanol (HFIP, Sigma-Aldrich) solvents to obtain a final concentration of 19.2% (w/v) and 4.8% (w/v) used as shell solution. SF with the concentration of 7.2% (w/v) was used as the core solution. The concentrations of PLCL (19.2%, w/v) and PEO (4.8%, w/v) were selected as the shell solution. 160 ng FGF-2/1.6 µg BSA was added to 1 ml shell solution; 1.6 µg CTGF/0.8 µg BSA was added to 0.2 ml core solution. For characterization of produced coaxial fibers by fluorescence imaging, 1 mg Tetramethyl rhodamine Bovine Serum Albumin (BSA) (Life technologies, Carlsbad, CA) was added to 1 ml core solution with SF and 1 mg Fluorescein isothiocyanate (FITC)-BSA (Sigma) was added to 1 ml shell solution with PLCL-PEO.

The coaxial needle consists of an inner needle with a diameter of 0.7 mm and an outer needle with a diameter of 1.8 mm. A high voltage power supply was applied between the coaxial needle and a grounded rotary drum collector. The core solution was injected at a controlled flow rate of 0.20 ml/h and the shell solution at 1.0 ml/h. The distance between the needles and the collector was set to 13 cm. All of the electrospun fibers were obtained at room temperature with a relative humidity of 37–45%. The obtained fibers were dried under vacuum (Labconco Freezone TriadTM) overnight to remove the excess solvents before further use. The fibers were then punched out as 6 mm Ø (~0.3 cm^2^) or 12 mm Ø (~1.1 cm^2^) scaffolds.

### Characterization of Fibers by Scanning Electron Microscopy, transmission electron microscopy and fluorescence microscopy

The morphologies of the electrospun fibers were examined with a high-resolution scanning electron microscopy (SEM) (FEI, Nova 600 NanoSEM). The fibers were placed directly into the SEM chamber without any metal sputtering or coating. All the images were captured using a secondary electron detector with an acceleration voltage of 5 kV under low vacuum conditions.

The core-shell structure of the SF/PLCL fibers was characterized by Transmission electron microscopy (TEM, Tecnai G2 Spirit, FEI Co.). The specimen was prepared by direct deposition of the electrospun fibers onto a carbon film coated copper grid (Ted Pella, Inc., Redding, CA). Images of SF/PLCL fibers were obtained at 120 kV.

To confirm the presences and distribution of TetramethylrhodamineBSA in the core of fibers and FITC-BSA in the shell of fibers, observations of Silk-TetramethylrhodamineBSA/PLCL-PEO-FITC-BSA fibers deposited on microscope glass slides were performed on a Zeiss LSM 700 laser confocal microscope (Carl Zeiss Micro-Imaging GmbH, Germany) and EVOS FL Auto Cell imaging system (Life Technologies, Carlsbad, CA, USA).

### Water Contact Angle Analysis

Water contact angle measurements were used to explore the surface wettability of the electrospun scaffold. All images of distilled water dropped onto the electrospun scaffolds were recorded using a Drop Shape Analyzer DSA100 (KRUSS GmbH, Hamburg, Germany), the images of the droplets on the scaffolds after 100 s were visualized and analyzed. The reported contact angle is the average of 3 measurements from different positions.

### Attenuated Total Reflection (ATR) Fourier Transform Infrared (FTIR) Spectroscopy

The ATR-FTIR spectra were recorded in the region of 4000–500 cm^−1^ using a Vertex 70 V vacuum spectrometer (Bruker) equipped with a diamond ATR crystal and a Deuterated Lanthanum α Alanine doped TriGlycine Sulphate (DLaTGS) detector. The resolution was set at 2 cm^−1^ and total of 64 scans were averaged to obtain one spectrum. The measurements were repeated at different locations on all the samples.

### *In Vitro* Release Profile

For *in vitro* release study, composite electrospun fibrous mats with FGF-2 loaded in shell and CTGF loaded in the core were tested. Each mesh weighing about 35 mg was soaked in 1 ml culture medium. The fibrous mats were incubated at 37 °C in a humidified atmosphere of 5% CO_2_. At specified time points, the release medium was completely removed and stored at −20 °C for analysis, and 1 ml of fresh medium was replenished for continuing incubation. The amount of FGF-2 and CTGF released from samples were quantified using the human FGF- 2 ELISA kit (Peprotech Nordic, Sweden) and human CTGF ELISA kit (Peprotech Nordic, Sweden) respectively. The release of FGF-2 and CTGF were quantified up to 14 days and the results were presented in terms of accumulative release percentage as a function of release time, all samples were run in triplicates.

### Cell Culture and Cell Seeding on Scaffolds

Rat mesenchymal stem cells from bone marrow (rMSCs) (Lonza, Switzerland) were purchased with their mesenchymal characteristics verified. These rMSCs from bone marrows are positive for surface markers CD29 and CD90, and negative for CD11b, CD34, and CD45. Cultures were maintained in rat mesenchymal stem cell growth medium (MSCGM) containing basal medium (MSCBM) supplemented with 10% mesenchymal stem cell growth supplement, 100U/ml gentamycin/ampicillin and 10% L-Glutamate (Lonza). Cells were grown at 37 °C in a humidified atmosphere of 5% CO_2_. Cells were enzymatically treated with Trypsin for passaging every 5–7 days. Passage 3 to 5 of rMSCs were used for cell differentiation experiment.

Cells were seeded and cultured with growth medium onto each group of samples at a density of 2 × 10^4^ cells cm^−2^ in 48-well plates. For the cells seeded on 4 groups of scaffolds, cells were kept in MSCGM and differentiated for a 2-week period, with conditioned medium change every third day.

Control experiments with cells cultured in differentiation media were conducted for a 2-week period. Osteogenic medium is consisting of DMEM-high glucose (Gibco, Life Technologies, Waltham, MA, America), 10% (v/v) FBS, 290 nM ascorbic acid-2-phosphate (Sigma Aldrich, America), 5 mM β-glycerophosphate (Sigma Aldrich), 100 nM dexamethasone (Sigma Aldrich) and 1% (v/v) penicillin/streptomycin (Gibco, Life Technologies). Chondrogenic differentiation medium is consisting of DMEM-high glucose (Gibco, Life Technologies, Waltham, MA, America), 10% (v/v) FBS, 1 µM ascorbic acid-2-phosphate (Sigma Aldrich, America), 100 nM dexamethasone (Sigma Aldrich) with 10 ng/ml TGFβ-3 (Life Technologies) and 1% (v/v) penicillin/streptomycin (Gibco, Life Technologies).

### Cytotoxicity Test (lactate dehydrogenase (LDH) activity)

After 24 hours of cell seeding, the LDH activity in the collected culture media was taken as an indicator of damaged cells. The activity of the cytosolic enzyme was estimated according to the manufacturer’s kit instructions (Roche Diagnostics, Mannheim, Germany), by assessing the rate of oxidation of NADH at 490 nm in the presence of pyruvate. Results from all the samples were presented relative to the LDH activity in the medium without cells cultured on TCP (low control, 0% of cell death) and of cells cultured on TCP treated with 0.1% Triton X-100 (high control, 100% cell death). The percentage of LDH activity was calculated using the equation 1:

1 $${\rm{Cytotoxicity}}\,( \% )=\frac{\exp .\,{\rm{value}}-{\rm{low}}\,{\rm{control}}}{{\rm{high}}\,{\rm{control}}-{\rm{low}}\,{\rm{control}}}\,\times \,100$$

### Live/Dead Staining

To analyze the viability of the cells, a cell viability kit (Ready Probes, Thermo Fisher Scientific) was used. One day after the cell seeding, NucBlue and NucGreen for all cells and dead cells respectively were introduced to the cell culture media. Images were taken after 15 min of incubation with the dyes, using a microscope, EVOS FL Auto (Thermo Fisher), at 20x magnification. Acquired images were analyzed using ImageJ software (NIH). Randomly selected areas (n ≥ 3) were used to count the number of live and dead cells (n ≥ 200).

### Cell Viability/Proliferation

Cell viability and proliferation of 7 days’ culture was assayed with Cell Counting Kit-8 (CCK-8, Dojindo, Kumamoto, Japan). Briefly, cell culture medium was removed, 4 groups of scaffolds were immersed with a diluted CCK-8 solution (20 µl of CCK-8 reagent in 200 µl fresh cell culture medium) in each well and incubated for 1 hour. The same volumes of culture medium and CCK-reagent without cells also incubated as the background. 100 µl of the solution was transferred into a 96-well plate, and the absorbance at 450 nm was measured for each well as above. The cell proliferation was examined after incubation on day 1, 3, 7 and 14, sextuplicate exposures of each sample were made under the same conditions.

### Real-time PCR

Total RNA was isolated using TRIzol (Sigma Aldrich, Schnelldorf, Germany) according to the manufacturer’s protocol. The resulting total RNA quantified using a Nanodrop ND-1000 (NanoDrop Technologies, Inc.) with OD260/280 nm ratios between 1.8 and 2.0 were used for cDNA synthesis, and stored at −80 °C prior to reverse transcription. 800 ng RNA was reverse transcribed to cDNA at 37 °C for 60 min using High Capacity RNA-to-cDNA kit (Applied Biosystems, Foster City, CA). For mRNA quantification, real-time quantitative PCR reactions with the cDNA samples were performed in a Lightcycler 480® (Roche Diagnostics, Mannheim, Germany) using SYBR green (Roche) detection with primers listed in Table 1. Fold differences were calculated using the standard ∆∆Ct method with GAPDH as the housekeeping. In PCRs with efficiencies approaching 100%, the amount of internal reference gene relative to a calibrator (fold change between two Ct values) is given by the equation 2:

2 $${\rm{Fold}}\,{\rm{difference}}={2}^{-{\rm{\Delta }}{\rm{\Delta }}\text{Ct}}$$

**Table 1:** Primers sequences used in the qPCR.

Gene	Primer Sequence (5′-3′)	Tm (°C)
*FSP1*	Forward AGGACAGACGAAGCTGCATT	58.5
	Reverse CTCACAGCCAACATGGAAGA	58.2
*COL1*	Forward TTGACCCTAACCAAGGATGC	58.4
	Reverse CACCCCTTCTGCGTTGTATT	58.5
*VIMENTIN*	Forward ACGAGTACCGGAGACAGGTG	58.6
	Reverse TCCAGCAGCTTCCTGTAGGT	58.5
*COL2*	Forward TCCTAAGGGTGCCAATGGTGA	63.4
	Reverse AGGACCAACTTTGCCTTGAGGAC	63.6
*OC*	Forward ATGCCACTGCGTATTGGTTGA	62.2
	Reverse TCCGCTAGCTCGTCACAATTG	62.2
*AP2*	Forward GACCTGGAAACTCGTCTCCA	58.7
	Reverse CATGACACATTCCACCACCA	59.7

### Immunocytochemistry

Cellular morphology was visualized at week1 and week2 using fluorescence microscopy. Briefly, cells and cell-laden constructs were fixed with 4% paraformaldehyde (PFA) in PBS (pH7.4) for 10 min at room temperature (RT). After rinsing with PBS for three times, the samples were placed in a permeabilization solution with 0.1% (v/v) Triton X-100 for 10 min and rinsed again with fresh PBS for three times. The cells and constructs were then blocked with 1% BSA in PBS for 1 hour at RT. Immunostaining with primary antibody rabbit monoclonal FSP1 (Abcam, Cambridge, UK) (1:400) and Alexa Fluor 647-conjugated donkey anti-rabbit IgG (Life technologies, Carlsbad, CA) (1:1000) was performed at 4 °C with gentle shaking for overnight, then counterstained with Phalloidin-Atto 488 and Hoechst 33258 (Life technologies, Carlsbad, CA) to visualize the f-actin and nuclei, respectively. The cells and cell-laden constructs were visualized using a Zeiss LSM 700 laser confocal microscope (Carl Zeiss Micro-Imaging GmbH, Germany).

### Statistical Analysis

Data are presented as the means ± standard error of mean. The statistical analyses were performed using Student’s *t*-test or a one-way repeated-measure Analysis of Variance test (ANOVA) to compare multiple data groups, followed by Turkey’s post hoc test. Values of p < 0.05 were considered statistically significant. The software used for statistics and the creation of figures was Prism 5 for Windows (GraphPad, San Diego, CA, USA).

## Results

### Characterization of electrospun SF/PLCL-PEO coaxial nanofibrous scaffolds

**Figure 1:**
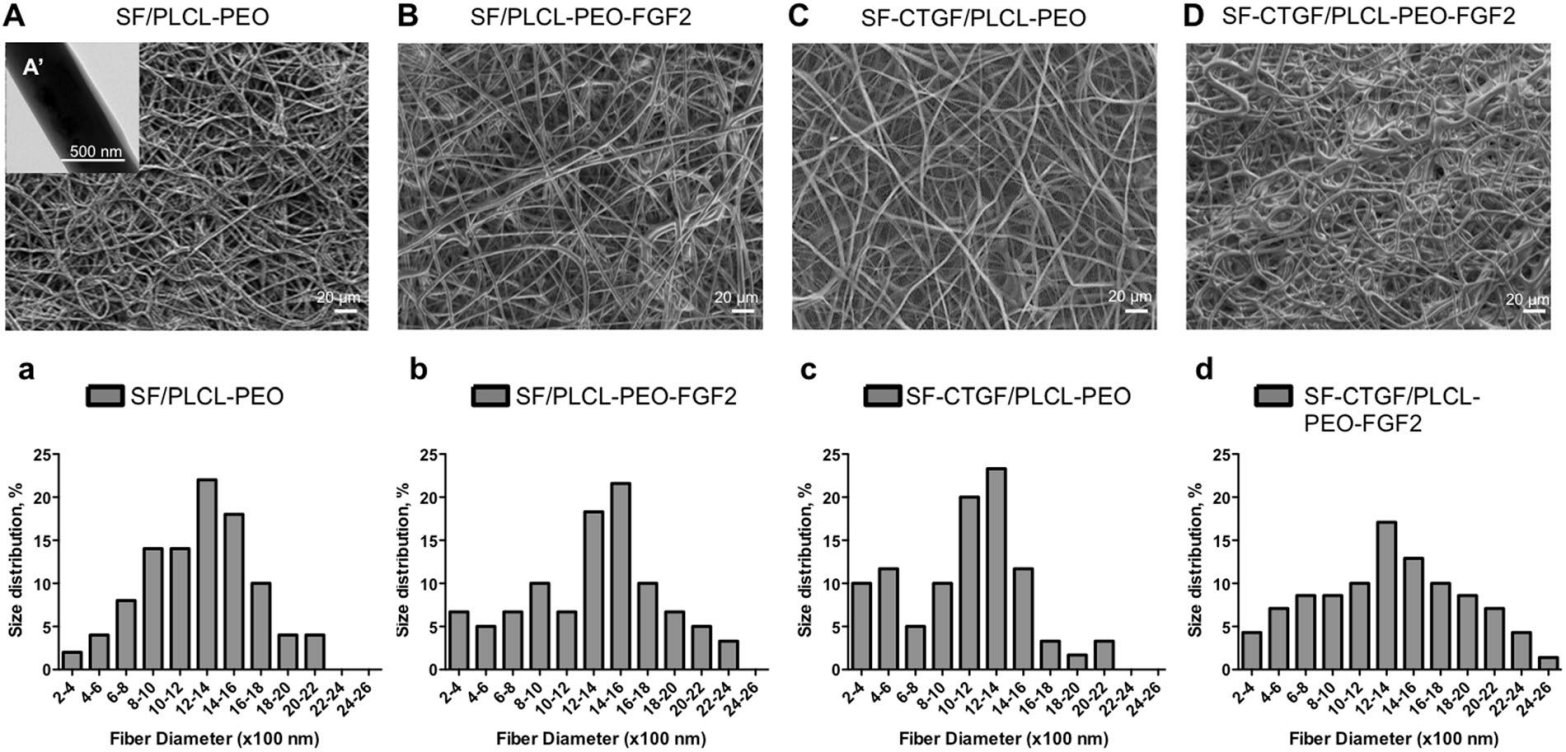
Characterization of coaxial electrospun scaffold. (**A**–**D**) SEM images of electrospun nanofibrous scaffolds. (A’) TEM image of the SF/PLCL-PEO coaxial fibers. (a–d) Fiber diameter distribution of four groups of scaffolds.

Coaxial electrospinning with co-occurrence of core and shell solutions, each dispensing at defined flow rates, requires preliminary experiments to adjust both electrospinning parameters (voltage, flow rates, nozzle-to-collector distance etc) and solution parameters (viscosity/concentration, solvents etc) for optimizing the electrospinning stability and fiber morphology (Fig. S1). Keeping electrospinning parameters constant and SF with a concentration of 7.2 w/v%, varying PLCL from 13 to 20 w/v% was able to render the particle morphology to fiber morphology, attributed to the increased solution viscosity. Further adding PEO to the PLCL shell solution, SF/PLCL-PEO fibers were successfully electrospun under stable processing conditions using PLCL (19.2%, w/v) and PEO (4.8%, w/v) as the final shell solution and 7.2% SF as the final core solution, described in Section 2.2. The SEM images show that the resultant fibers SF/PLCL-PEO (diameter 1.19 ± 0.34 µm), SF/PLCL-PEO-bFGF (diameter 1.43 ± 0.41 µm), SF-CTGF/PLCL-PEO (diameter 1.36 ± 0.23 µm) and SF-CTGF/PLCL-PEO-bFGF (diameter 1.57 ± 0.47 µm) appeared to be bead-free and randomly arrayed (Fig. 1). The TEM image in Fig. 1A’ clearly demonstrated the core-shell structure of SF/PLCL-PEO.

**Figure 2:**
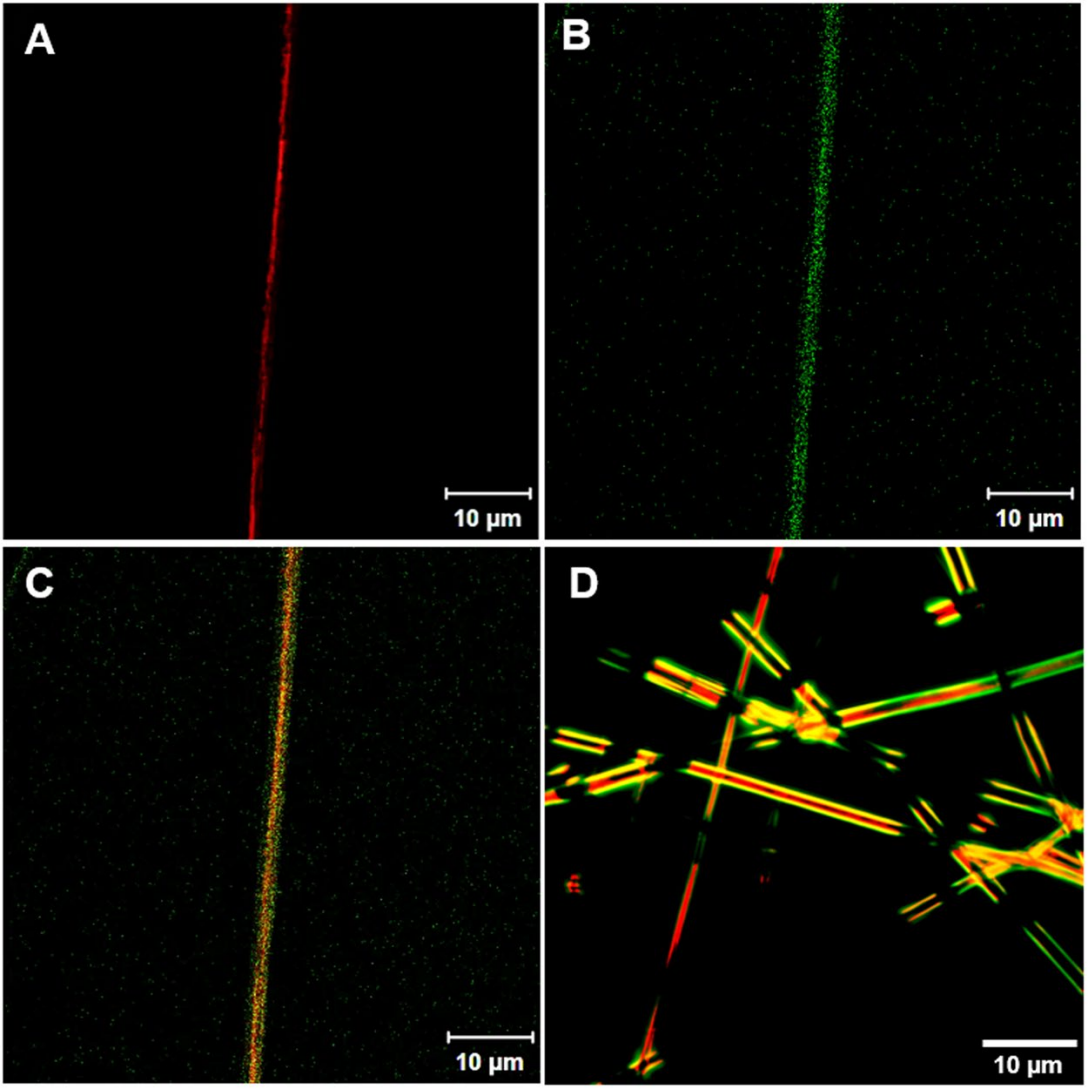
Microscope images of coaxial electrospun fibers. (**A**–**C**) Confocal images of coaxial fibers with RhodamineBSA shown red fluorescence in the core (**A**) and FITC-BSA shown green fluorescence in the shell (**B**) respectively, and the merged image (**C**). (**D**) Phase contrast merged image showed the distribution of proteins in the core-shell structures with red fluorescence RhodamineBSA in core and green fluorescence FITC-BSA in shell.

As further evaluation of localization of proteins in the core-shell structure, fluorescence imaging demonstrated that the core emitted red fluorescence, suggesting the presence of Tetramethylrhodamine-labeled BSA protein loaded in the core, and the shell emitted green fluorescence, suggesting the presence of FITC-labeled BSA protein loaded in the shell (Fig. 2, S2).

**Figure 3:**
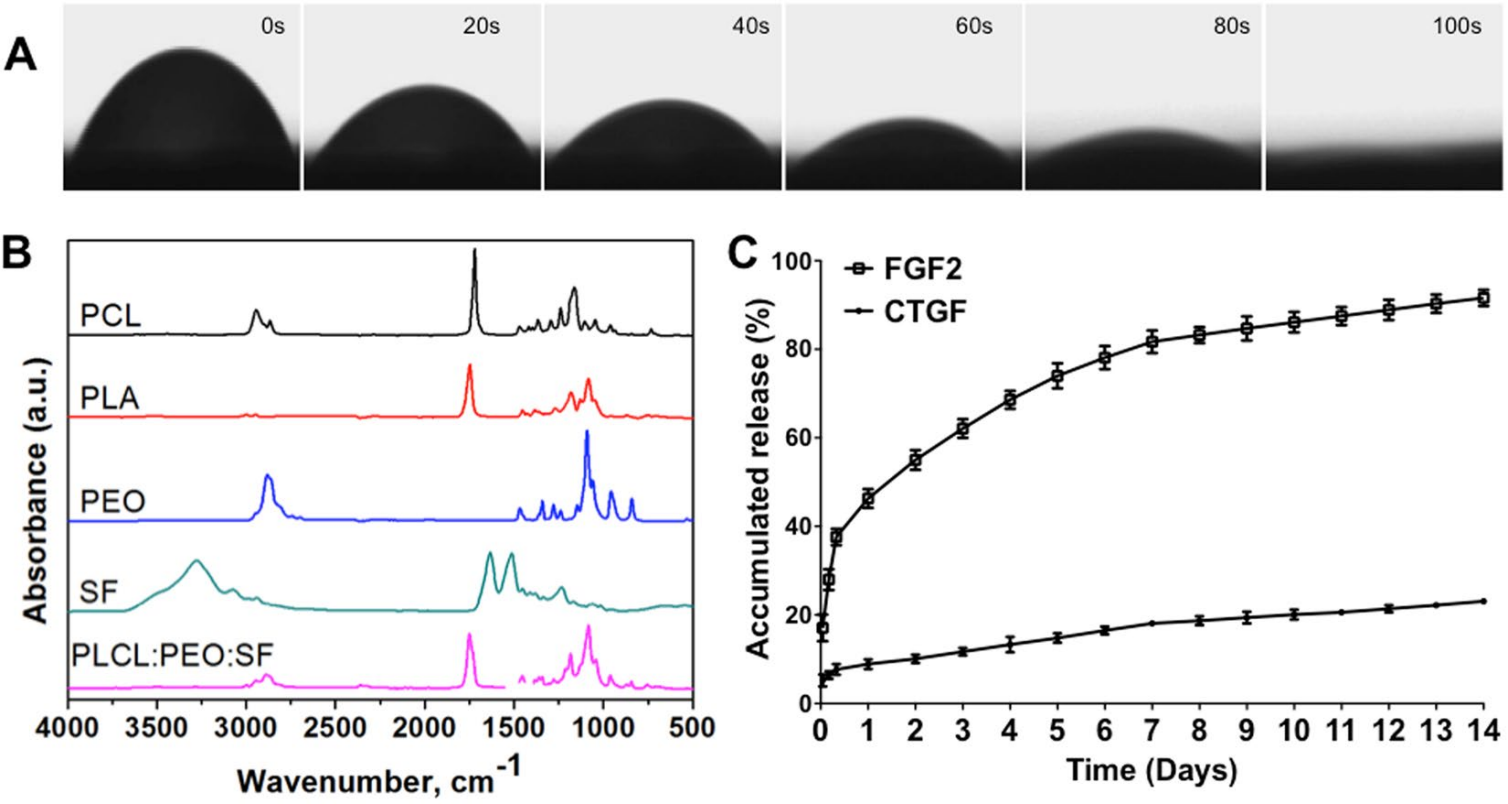
Physical property of scaffolds. (**A**) Representative images of the water contact angle (WCA) of the coaxial SF/PLCL-PEO fibers. (**B**) ATR-FTIR absorbance spectra of pure PLCL, PEO, SF and SF/PLCL-PEO fibers. (**C**) Release profile of FGF-2 and CTGF from the shell (•) and core (○) of coaxial scaffolds by ELISA assay, respectively.

The surface wettability of SF/PLCL-PEO was analyzed through water contact angles measurement and the fibers appeared to be hydrophilic, absorbing water quickly and presenting a water contact angle of 12.3 ± 1.3° at 100 s (Fig. 3A).

Attenuated total reflection (ATR) is used in conjunction with infrared spectroscopy to enable samples to be examined (with a penetration depth of 0.5–1.5 µm) directly in the solid or liquid state without further preparation. ATR-FTIR spectra of electrospun fibers of SF/PLCL-PEO along with individual components are shown in Fig. 3B. The two peaks at 2945 and 2888 cm^−1^ corresponded to asymmetric CH_2_ stretching *ν*_*as*_ (CH_2_) and symmetric CH_2_ stretching *ν*_*s*_ (CH_2_), respectively^29–31^. Carbonyl stretching *ν*(C = O) from PLCL was found at 1751 cm^−1^. The asymmetric deformation of CH_3_ and symmetric deformation of CH_3_ were found at 1454 cm^−1^ and at 1360 cm^−1^, respectively. Asymmetric stretching *ν*_*as*_(C-O-C) and twisting *τ*(CH_3_), symmetric stretching *ν*_*s*_(C-O-C), symmetrical stretching were found at 1183 cm^−1^ and 1084 cm^−1^, respectively. Moreover, the peaks appeared at around 1341 cm^−1^ (the CH2 wagging), 961 cm^−1^ (the symmetric CH2 rocking) and 1062 cm^−1^ (the COC asymmetric stretching and CH2 symmetric rocking), confirmed the presence of PEO at the fiber surface. On the other hand, the characteristic peaks of N-H stretching vibration (3380 cm^−1^), C-H stretching vibration (3100 cm^−1^), C = O stretching (1625 cm^−1^) C-N stretching and N-H in-plane bending (1513 cm^−1^) from SF^32, 33^ were not found, indicating that SF was well encapsulated in the core.

### Release profile of electrospun SF-CTGF/PLCL-PEO-FGF2 coaxial nanofibrous scaffolds

The release profile of growth factors from core and shell was studied by ELISA assay (Fig. 3C). With reference to the shell-loaded FGF-2, there was an initial burst release of 37.6 ± 1.8% within the beginning 8 h. Then, the release slowed down to around 5–8% of release per day in the first week. At 7d, the accumulated amount released reached 81.7 ± 2.6%. After day 7, the release profile exhibited a decelerating release rare of below 1% per day, and the accumulated amount of FITC-FGF-2 released from the shell reached 91.6 ± 1.8% at day 14. With reference to the CTGF that was incorporated in the core, much less burst was observed compared to FGF-2, resulting accumulatively 10.1 ± 0.9% of release was obtained at the beginning 3 days, which was followed with a sustained, steadily release, where the accumulative release at day 14 was only 23.1 ± 0.6%.

### Cytocompatibility and proliferation of rMSCs on SF/PLCL-PEO nanofibrous scaffolds

**Figure 4:**
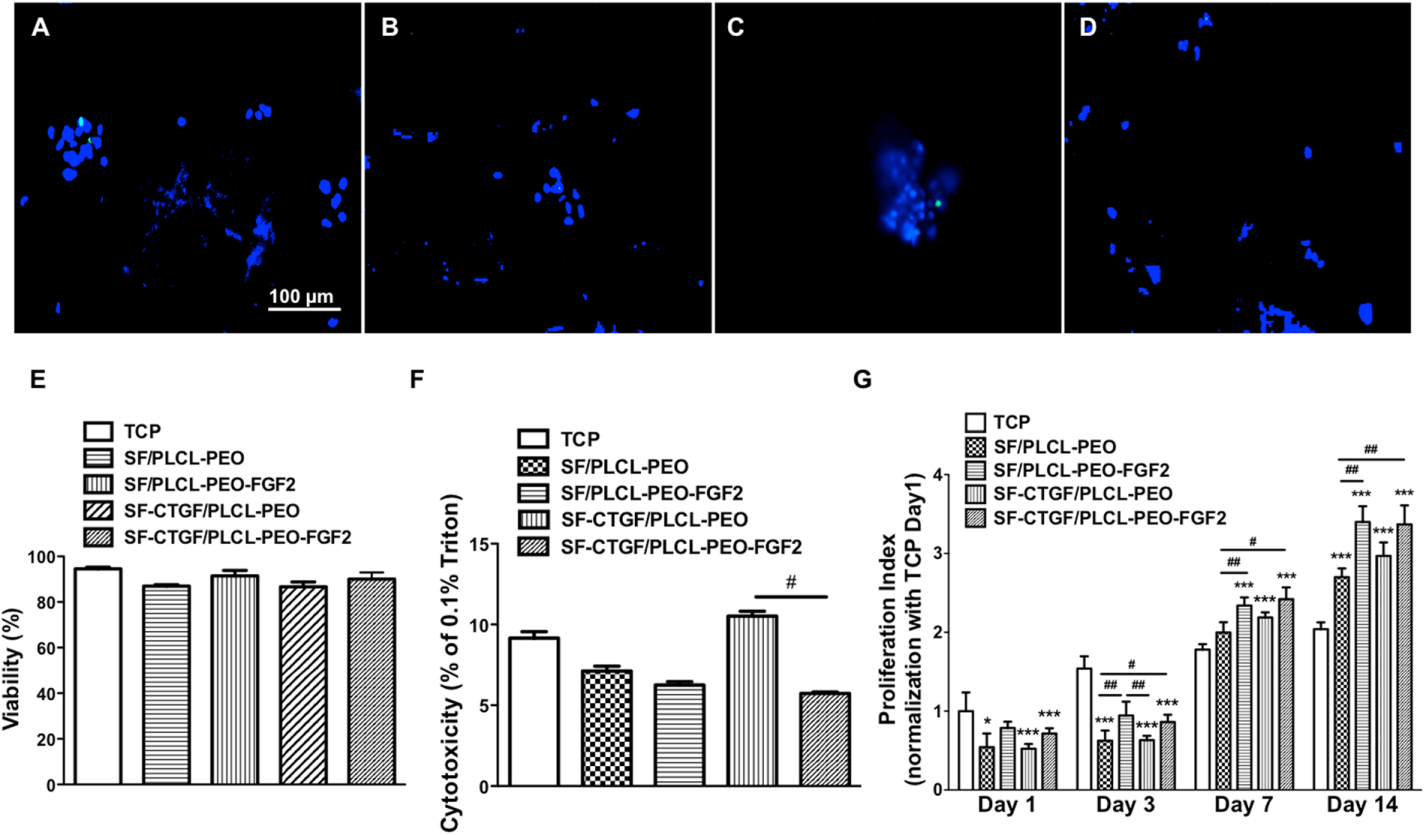
Analysis of biocompatibility of different coaxial scaffolds. (**A**–**D**) Representative images of live-dead staining of rMSCs after 1 day culture on different scaffolds SF/PLCL-PEO (**A**), SF/PLCLPEO-FGF2 (**B**), SF-CTGF/PLCL-PEO (**C**) and SF-CTGF/PLCL-PEO-FGF2 (**D**). All cells were counterstained with NucBlue live reagent, nuclei of dead cells were counterstained with NucGreen dead reagent. (**E**) The quantified data from (**A**–**D**), at least 200 cells were counted for each group. (**F**) Analysis of cytotoxicity of different scaffolds to rMSCs after 1 day culture by LDH assay. (**G**) Analysis of rMSCs proliferation on different scaffolds after a 14-day incubation by CCK assay. (One-way repeated-measure Analysis of Variance test (ANOVA) were used to compare multiple groups. * and *** stand for significant difference between samples on scaffolds and samples on tisue culture plastic (TCP) at same time point with p < 0.05 and p < 0.001, respectively; # and ## stand for significant difference among different scaffolds at same time point with p < 0.05 and p < 0.01, respectively).

To assess cytotoxicity of different scaffolds on rMSCs, live/dead staining and LDH assay were performed. Cell viability was visualized by live/dead staining after 1 day culture on the scaffolds (Fig. 4A–D). It is shown that rMSCs were adhered and spread on the scaffolds, except that group of SF/PLCL-PEO and SF-CTGF/PLCL-PEO presented cluster cells, which could be attributed to the non-adherent nature of PEO; while the groups containing FGF-2 showed improved spreading, which is consistent with the literature^34^.

The number of live and dead cells were counted, and a viability of ~85% was observed with no statistical differences among all groups, confirming the biocompatibility of all the groups (Fig. 4E). In parallel, LDH overall values around 10% with no significant differences between these four scaffolds and TCP confirmed that all scaffolds provide biocompatible environments for cells. Interestingly, a significant difference was found between SF-CTGF/PLCL-PEO and SF-CTGF/PLCL-PEO-FGF2, suggesting the important role of FGF-2 in the initial cell adhesion and spreading which is consistent with the literature^34^ (Fig. 4F).

To further evaluate the effect of nanofibrous scaffolds on rMSCs proliferation, CCK-8 assay was conducted at day 1, 3 7 and 14 (Fig. 4G). At both day 1 and day 3, significantly lower values were observed among the fiber groups compared to TCP, except the group of SF/PLCL-PEO-FGF2 showed comparable value to TCP. At both day 7 and day 14, cells on both SF/PLCL-PEO-FGF2 and SF-CTGF/PLCL-PEO-FGF2 showed significantly higher proliferation than that of SF/PLCL-PEO, confirming FGF-2’s positive role in MSCs’ survival and proliferation. No obvious proliferation was observed for all fiber groups of scaffolds at day 3 compared to day 1; while significantly higher proliferation rates were found on all fiber groups except SF/PLCL-PEO compared to TCP at day 7; at day 14, all the fiber groups supported faster proliferation than TCP, confirming the 3D cell culture environment. Besides the lower cell confluency upon seeding on 3D fibers, the delayed proliferation compared to TCP could be attributed to PEO as non-adherent substrate.

### Effect of electrospun SF/PLCL-PEO nanofibrous scaffolds on rMSCs differentiation

**Figure 5:**
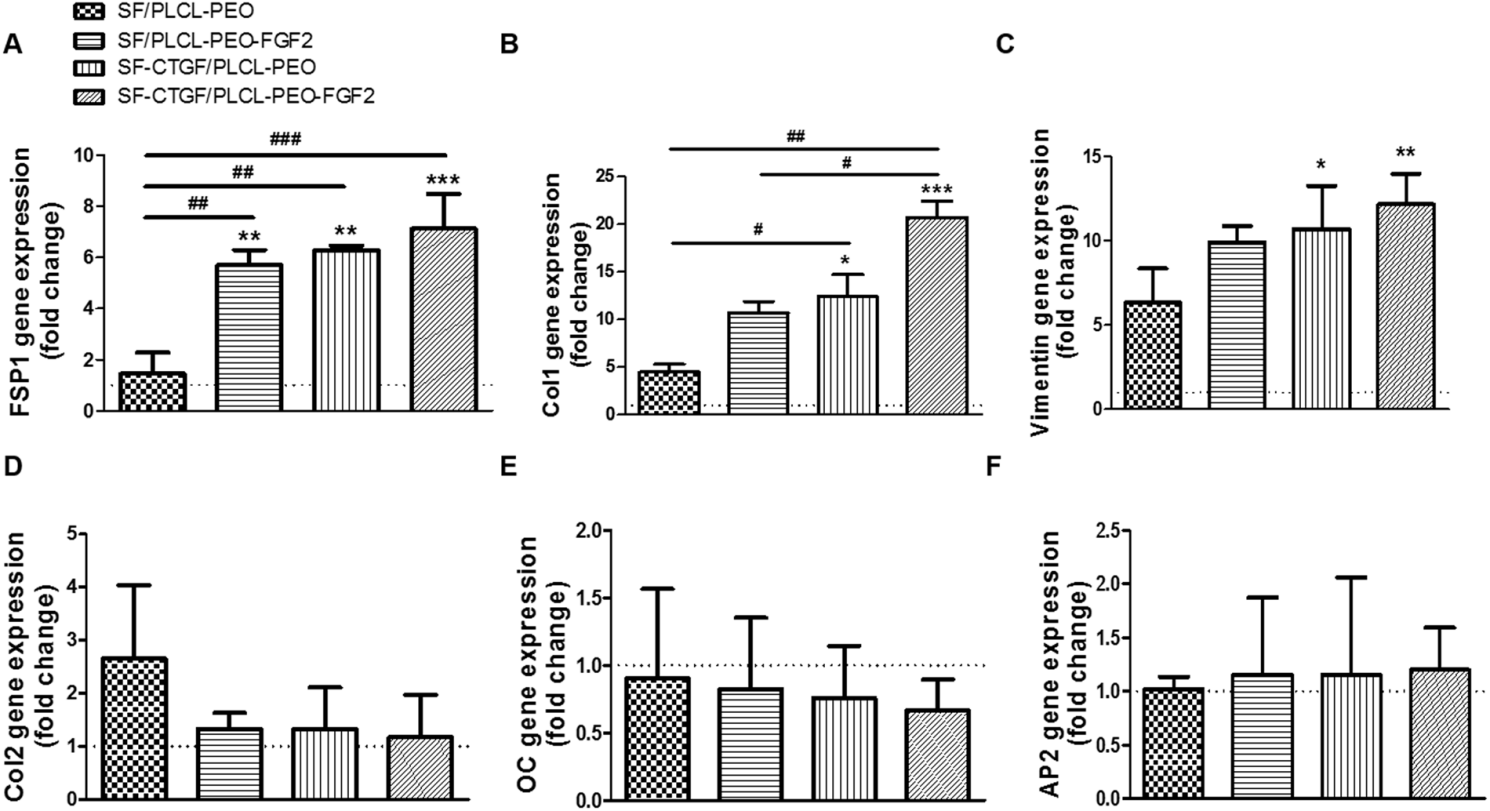
Quantitative gene expression levels of (**A**) FSP1 (a marker of fibroblast), (**B**) COL1 (a marker of fibroblasts), (**C**) VIMENTIN (a marker of fibroblasts), (**D**) COL2 (a marker of chondrocytes), (**E**) OC (a marker of osteoblasts) and (**F**) AP2 (a marker of adipocytes) in the cells cultured on different coaxial nanofibrous scaffolds compared with the levels observed on the TCP at week 1. (One-way repeated-measure Analysis of Variance test (ANOVA) were used to compare multiple groups. *, ** and ***: stand for significant difference between samples on scaffolds and samples on TCP with p < 0.05, p < 0.01 and p < 0.001, respectively; #, ## and ###: stand for significant difference among different scaffolds with p < 0.05, p < 0.01 and p < 0.001, respectively).

To understand the influence of four groups of scaffolds on rMSCs differentiation, gene and protein expressions of cells were evaluated through qPCR and immunocytochemistry. The RNA expression levels of fibrogenic markers FSP1, COL1 and VIMENTIN were tested after 1 week cell culture on the scaffolds, along with chondrogenic marker COL2, osteogenic marker OC and adipogenic marker AP2. The qPCR results show that the RNA expression levels of FSP1 were significantly higher in the groups containing GFs, especially SF-CTGF/PLCL-PEO-FGF2 (with P < 0.005), than that in the group of SF/PLCL-PEO (Fig. 5A). In addition, cells on CTGF loaded groups, particularly SF-CTGF/PLCL-PEO-FGF2 (with P < 0.005), exhibited a marked up-regulation of COL1 and VIMENTIN expression in comparison to cells on SF/PLCL-PEO, (Fig. 5B,C). In parallel, the expression levels of COL2, OC and AP2 in all groups were comparable to or lower than (with no significances) those in the group of TCP (Fig. 5D–F). In comparison, control experiments of rMSCs under chondrogenic, osteogenic and adipogenic induction showed their retained multi-potency (Fig. S3), demonstrating SF-CTGF/PLCL-PEO-FGF2 accelerates the differentiation of rMSCs into fibroblasts while alleviates chondrogenesis, osteogenesis and adipogenesis.

**Figure 6:**
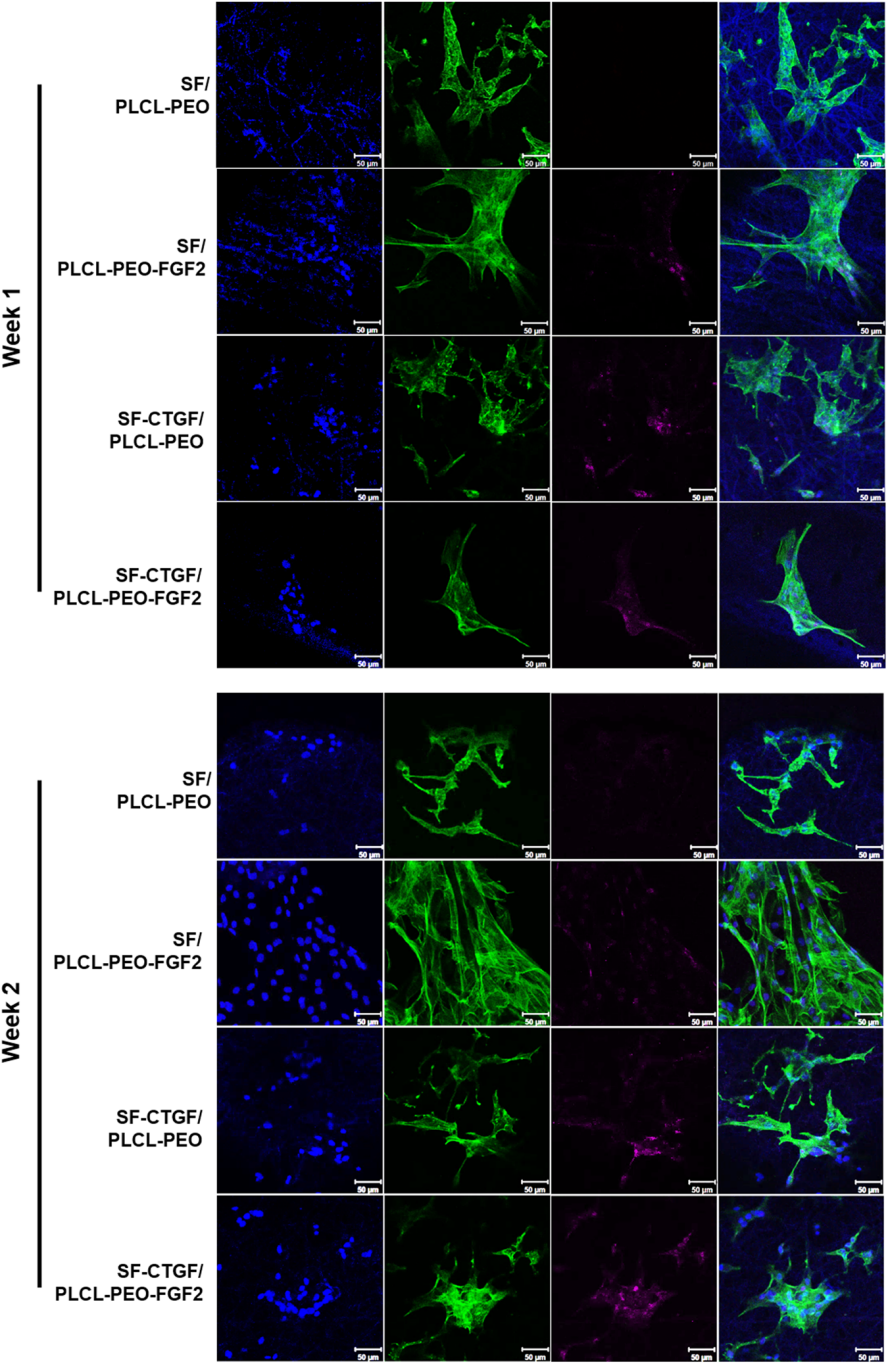
Immunofluorescence of fibroblastic differentiation for rMSCs on different coaxial nanofibrous scaffolds. (**A**) After 1 week culture on different scaffolds. (**B**) After 2 week culture on different scaffolds. Cells were stained for nuclei (blue), F-actin (green) and FSP1 (violet).

The immunocytochemistry analysis (Fig. 6) shows that cells on the SF-CTGF/PLCL-PEO-FGF2 expressed significantly higher amount of FSP1, suggesting the synergy of the dual-delivered CTGF and FGF-2 on fibroblastic differentiation of MSCs, which is also consistent with the qPCR results (Fig. 5A).

## Discussions

Coaxial electrospinning or core-shell electrospinning using two concentric needles is a sophisticated technology for the fabrication of scaffolds that can provide the sustained release of incorporated growth factors^35, 36^. The core of coaxial electrospinning facilitates the incorporation and preservation of bioactive substances^37^, whereas the shell was often used to protect sensitive substances encapsulated in the core. In our study, it is for the first time reported that a dual-delivery system, using coaxial electrospun fibers of PLCL/PEO/BSA/FGF2 solution as the shell fluid and SF/BSA/CTGF solution as the core fluid, could both enrich MSCs and direct their fibrogenic differentiation.

Silk Fibroin (SF) provides interesting biomaterials for tissue engineering and drug delivery system due to their non-immunogenicity, biocompatibility, and biodegradability^38^. They have been demonstrated to be efficient in delivering BMP-2^39^ and nerve growth factor (NGF)^40^ with the controlled release kinetics for MSC chondrogenesis and neuron regeneration, respectively. Here, SF was used to deliver CTGF from the core of the coaxial fibers. BSA, co-encapsulated with either FGF-2 or CTGF, functions as a protective agent and protein stabilizer to preserve growth factors^41^. The shell of electrospun coaxial fibers, on the other hand, was composed of PLCL and PEO. PLCL is a hydrophobic biodegradable polymer. Deploying PLCL as the shell will serve a diffusion barrier for the CTGF incorporated in the SF core, which would consequently slow down the release of CTGF. Hydrophilic PEO was added to hydrophobic PLCL to not only speed up the diffusion process for FGF-2 release from the shell, but also suppress protein adhesion for creating an inert cell-material interface for evaluating the effects of the released growth factors.

A temporarily sequential release of FGF-2 and CTGF were successfully achieved in this manner (Fig. 3C). The release mechanism is a diffusion/dissolution-controlled, matrix/reservoir combinatory system. FGF-2 is embedded in a dissolving matrix made of PLCL and PEO, where PEO is water-soluble (will gradually dissolve) and PLCL is hydrolytic (will gradually degrade). The release of FGF-2 is dissolution controlled; the dissolution rate depends on the molecular weight of PEO (the smaller, the faster), composition of monomer L/CL ratio in PLCL (the more of L, the faster) and the rate of dissolution fluid penetration into the matrix, which can be controlled by the ratio between PEO and PLCL (the more PEO, the faster dissolution fluid penetration). CTGF is embedded in SF matrix core and then further entrapped in the PLCL-PEO shell as a matrix/reservoir combinatory system. The release rate of CTGF is both dissolution and diffusion controlled, which depends on the water penetration through the PLCL/PEO shell (the thinner, the faster) and the SF core (the more β-sheet composition, the slower) to enable CTGF diffuse through the swollen network into the external environment. Consequently, unlike a burst release of FGF-2, CTGF shows a rather prolonged steady release.

The burst released FGF-2 from the shell was capable of enhancing initial MSC cell adhesion and spreading (Fig. 4A–D) which is consistent with previous studies^42^. Better initial adhesion and spreading could have important significance for tissue engineering, which could affect the subsequent bioactivity of cells on scaffolds^43^, and this is further proven by the proliferation assay (Fig. 4G).

Here, our hypothesis is that after the enrichment of MSCs under stimulation of burst released FGF-2, the sequentially released CTGF from the core would direct MSCs’ fibrogenic differentiation. CTGF contains the four modules that are characteristic of the CCN protein family (growth factor binding, integrin recognition, heparin/proteoglycan interaction(s), and a dimerization) and resembles domains within ECM proteins. It is therefore defined as a matricellular protein that bridges the functional division between structural extracellular macromolecules to interact with integrins and growth factors (such as bone morphogenetic protein-4, transforming growth factor-β). Although the mechanism is not clearly understood yet, there is emerging evidence that it also acts as an essential mediator for skeletogenesis in development. Here, the sustained release of CTGF from the core effectively directed fibrogenic differentiation of MSCs (Figs 5 and 6) confirming its active role in MSC fibrogenesis^17^.

Apart from maintaining MSCs proliferation potential, FGF-2 has also been reported with different effects on MSC fate regulations when accompanied with different growth factors^44^, such as a continuous or high concentration of exogenous FGF-2 repressed bone-specific gene expression^45, 46^, whereas endogenous FGF-2 is essential for osteogenesis^7^. Here when accompanied with CTGF, FGF-2 has shown a slightly additive effect on fibrogenic differentiation of MSCs (Figs 5 and 6).

Collectively, in this study the dual-delivery of CTGF and FGF-2 in a core-shell nanofibrous vehicle synergistically enhanced enrichment and fibroblastic differentiation of rMSCs *in vitro*. Here rMSCs were used in order to facilitate our follow-up *in vivo* animal study for pelvic floor repair using a rat abdominal wall defect model. Indeed human MSCs could be more relevant and significant towards clinical translation, and immune-deficient animal models should be applied. Keeping in mind that the *in vivo* environment is far more complex, the release kinetics should be further optimized by modulating each elements in this specific matrix/reservoir combinatory system, including the molecular weight of PEO, the monomer ratio of PLCL, the ratio of PLCL and PEO and the flow rate of core and shell solution for adjusting the thickness and permeability of the shell, and the β-sheet composition of SF for tuning the affinity and permeability of the core.

## Conclusions

Overall, this study demonstrates that dual-delivery of CTGF and FGF-2 from a core-shell fiber of SF/PLCL-PEO enhanced fibrogenic lineage differentiation of MSCs. The core-shell structure was confirmed by fluorescence microscopy and ATR-FTIR. Burst release of the FGF-2 and sustained release of CTGF were successfully achieved in this manner. FGF-2 plays an important role in stem cell survival and proliferation and, meanwhile when accompanied with CTGF, has a slightly additive effect on fibrogenic differentiation of MSCs, whereas CTGF promotes fibrogenesis and inhibits osteogenesis, chondrogenesis and adipogenesis. The work should provide instructive insights towards the improvement of stem cell transplantation for connective tissue regeneration.

## Electronic supplementary material

Supplementary materials
