# Supplementary material for: Dual-delivery of FGF-2/CTGF from Silk Fibroin/PLCL-PEO Coaxial Fibers Enhances MSC Proliferation and Fibrogenesis
Source: Sci Rep. 2017 Aug 17;7:8509. doi: 10.1038/s41598-017-08226-0 (PMC5561253; doi:10.1038/s41598-017-08226-0)
Supplement: Supplementary file 1 — Supplementary materials [file 41598_2017_8226_MOESM1_ESM.pdf]

Supplementary Materials

Dual-delivery of FGF2/CTGF from Silk Fibroin/PLCL-PEO Coaxial Fibers Enhance MSC Proliferation and Fibrogenesis

Ruodan Xu,[a] Huiling Zhao,[b] Hanif Muhammad,[b] Mingdong Dong,[b] Flemming Besenbacher[b] and Menglin Chen[a,b]*

[a] *Department of Engineering, Aarhus University, DK-8000 Aarhus C, Denmark*

[b] *Interdisiplinary Nanoscience Center (iNANO), Aarhus University, DK-8000 Aarhus C, Denmark*

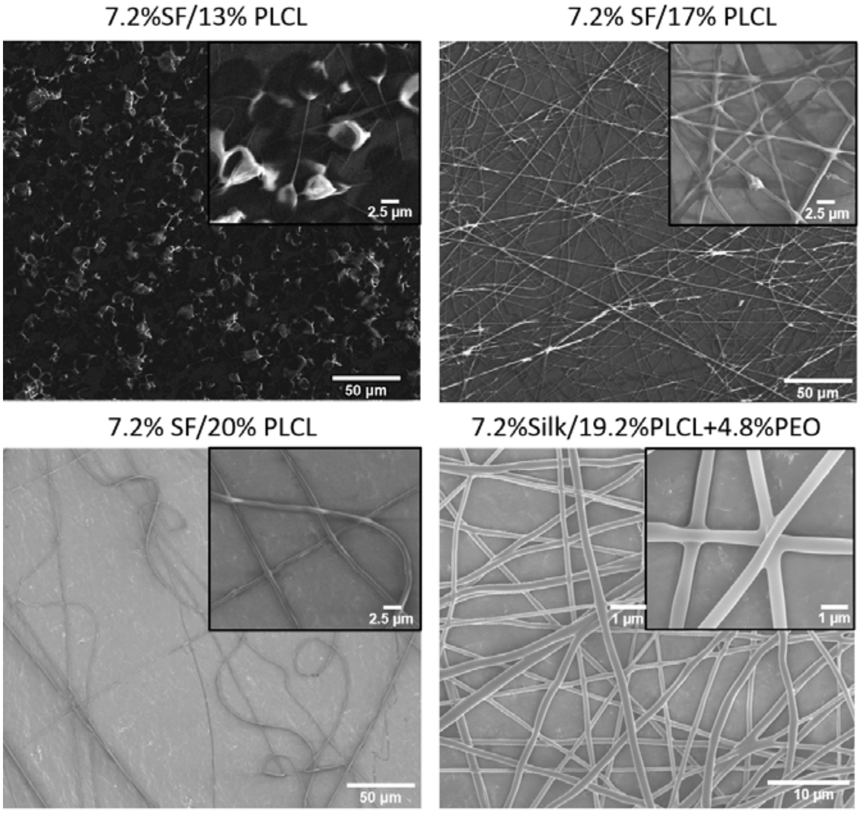

Figure S1 SEM images of core-shell SF/PLCL-EPO fibers at different concentrations.

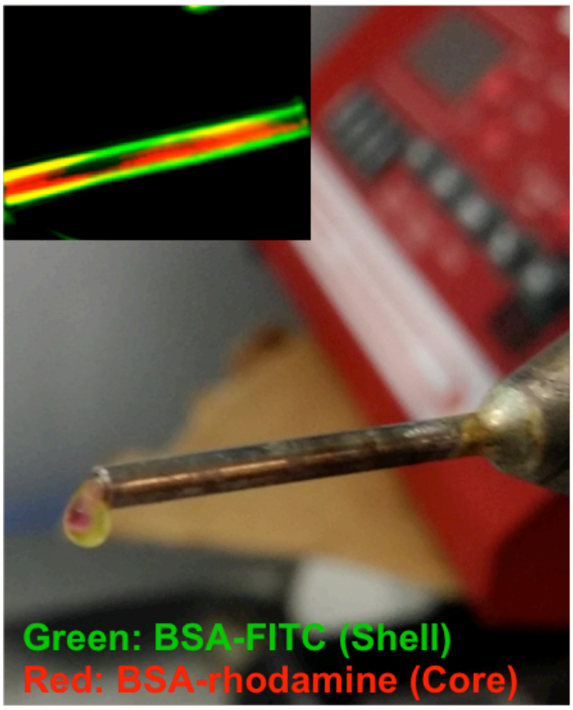

Figure S2 The core-shell solutions and resulted fibers with different dyes in the core and shell.

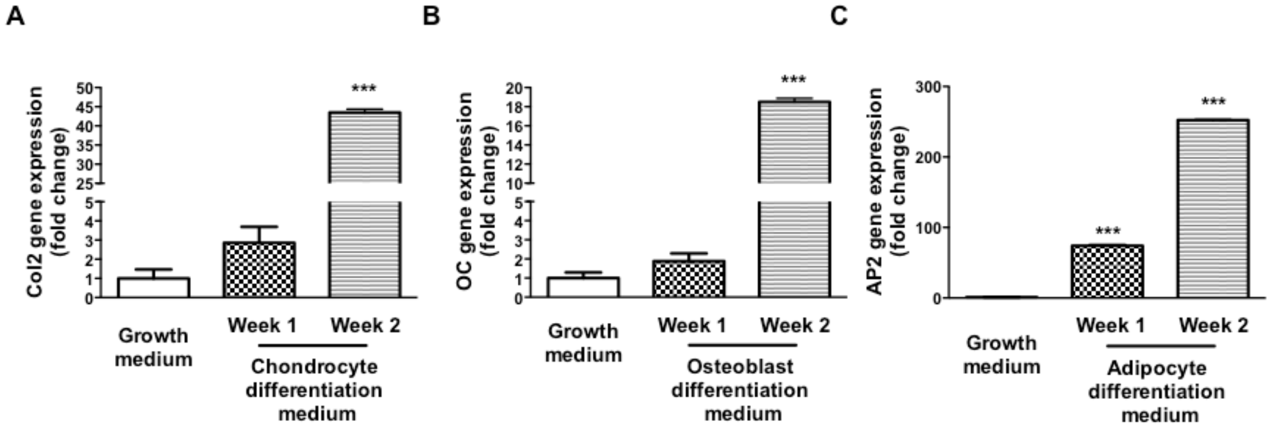

Figure S3 Quantitative gene expression levels of (A) COL2 (a marker of chondrocytes), (B) OC (a marker of osteoblasts) and (C) AP2 (a marker of adipocytes) in the cells cultured in different differentiation media on SF/PLCL-PEO at week 1 and 2. (*** stands for significant difference compared to growth medium with $p<0.001$)
